# Competitive SARS-CoV-2 Serology Reveals Most Antibodies Targeting the Spike Receptor-Binding Domain Compete for ACE2 Binding

**DOI:** 10.1128/mSphere.00802-20

**Published:** 2020-09-16

**Authors:** James R. Byrnes, Xin X. Zhou, Irene Lui, Susanna K. Elledge, Jeff E. Glasgow, Shion A. Lim, Rita P. Loudermilk, Charles Y. Chiu, Taia T. Wang, Michael R. Wilson, Kevin K. Leung, James A. Wells

**Affiliations:** a Department of Pharmaceutical Chemistry, University of California, San Francisco, San Francisco, California, USA; b Weill Institute for Neurosciences, University of California, San Francisco, San Francisco, California, USA; c Department of Neurology, University of California, San Francisco, San Francisco, California, USA; d Department of Laboratory Medicine, University of California, San Francisco, San Francisco, California, USA; e Department of Medicine, University of California, San Francisco, San Francisco, California, USA; f Chan Zuckerberg Biohub, San Francisco, California, USA; g Department of Medicine, Stanford University Medical School, Stanford, California, USA; h Department of Microbiology and Immunology, Stanford University Medical School, Stanford, California, USA; i Department of Cellular and Molecular Pharmacology, University of California, San Francisco, San Francisco, California, USA; National Institute of Allergy and Infectious Diseases

**Keywords:** COVID-19, SARS-CoV-2, angiotensin-converting enzyme 2, immunoserology, neutralizing antibodies, receptor-binding domain, serology

## Abstract

As severe acute respiratory syndrome coronavirus 2 (SARS-CoV-2) continues to spread around the world, there is an urgent need for new assay formats to characterize the humoral response to infection. Here, we present an efficient, competitive serological assay that can simultaneously determine an individual’s seroreactivity against the SARS-CoV-2 Spike protein and determine the proportion of anti-Spike antibodies that block interaction with the human angiotensin-converting enzyme 2 (ACE2) required for viral entry. In this approach based on the use of enzyme-linked immunosorbent assays (ELISA), we present natively folded viral Spike protein receptor-binding domain (RBD)-containing antigens via avidin-biotin interactions. Sera are then competed with soluble ACE2-Fc, or with a higher-affinity variant thereof, to determine the proportion of ACE2 blocking anti-RBD antibodies. Assessment of sera from 144 SARS-CoV-2 patients ultimately revealed that a remarkably consistent and high proportion of antibodies in the anti-RBD pool targeted the epitope responsible for ACE2 engagement (83% ± 11%; 50% to 107% signal inhibition in our largest cohort), further underscoring the importance of tailoring vaccines to promote the development of such antibodies.

**IMPORTANCE** With the emergence and continued spread of the SARS-CoV-2 virus, and of the associated disease, coronavirus disease 2019 (COVID-19), there is an urgent need for improved understanding of how the body mounts an immune response to the virus. Here, we developed a competitive SARS-CoV-2 serological assay that can simultaneously determine whether an individual has developed antibodies against the SARS-CoV-2 Spike protein receptor-binding domain (RBD) and measure the proportion of these antibodies that block interaction with the human angiotensin-converting enzyme 2 (ACE2) required for viral entry. Using this assay and 144 SARS-CoV-2 patient serum samples, we found that a majority of anti-RBD antibodies compete for ACE2 binding. These results not only highlight the need to design vaccines to generate such blocking antibodies but also demonstrate the utility of this assay to rapidly screen patient sera for potentially neutralizing antibodies.

## Abstract (precis)

With the emergence and continued spread of the SARS-CoV-2 virus, and of the associated disease, coronavirus disease 2019 (COVID-19), there is an urgent need for improved understanding of how the body mounts an immune response to the virus. Here, we developed a competitive SARS-CoV-2 serological assay that can simultaneously determine whether an individual has developed antibodies against the SARS-CoV-2 Spike protein receptor-binding domain (RBD) and measure the proportion of these antibodies that block interaction with the human angiotensin-converting enzyme 2 (ACE2) required for viral entry. Using this assay and 144 SARS-CoV-2 patient serum samples, we found that a majority of anti-RBD antibodies compete for ACE2 binding. These results not only highlight the need to design vaccines to generate such blocking antibodies but also demonstrate the utility of this assay to rapidly screen patient sera for potentially neutralizing antibodies.

## INTRODUCTION

The emergence of severe acute respiratory syndrome coronavirus 2 (SARS-CoV-2) in humans, and the respiratory disease associated with infection, coronavirus disease 2019 (COVID-19), has placed a significant public health burden on countries worldwide. Viral entry is dependent on a binding interaction between the receptor-binding domain (RBD) of the viral Spike protein and angiotensin-converting enzyme 2 (ACE2) on the cell surface (1, 2). Given the crucial role of RBD binding to ACE2 in infection, disrupting this interaction has emerged as a promising target for first-generation biologics to provide passive immunity, either with anti-Spike antibodies (3) or with ACE2 constructs (4, 5). As more patients recover from SARS-CoV-2 infection, there is an increasing need for serology assays to examine the humoral response to infection and vaccination.

Although direct detection of viral proteins or PCR testing is key to diagnosing the early stages of SARS-CoV-2 infection, serological assays detecting anti-SARS-CoV-2 antibodies are vital tools to monitor the evolution of antiviral responses after the period of acute infection ends (6, 7). Serological assays take many forms, including enzyme-linked immunosorbent assays (ELISA) (8), viral neutralization assays, and rapid lateral-flow assays (9). Neutralization assays performed with serum necessitate culture of either live or pseudovirus, and rapid lateral-flow diagnostic tests provide heterogeneous results (10) that are difficult to quantify. ELISA-based serology tests provide quantitative results and are easily adapted to test a variety of conditions and experimental designs. One clear issue is that of whether a patient has developed antibodies in serum with neutralizing activity. A modified ELISA-type serology assay can rapidly screen for patient antibodies that compete with ACE2 for RBD binding and that therefore may disrupt RBD binding to ACE2 and block viral entry. Improved understanding of the prevalence of these antibodies in patient sera will inform both therapeutic and vaccine design efforts and will offer improved resolution with respect to the antibody pool found in convalescent-phase serum.

Here, we report the development of a simple competitive serological assay using biotinylated Spike protein antigens and a dimeric ACE2-Fc fusion construct. Use of the avidin-biotin interaction to coat plates with biotinylated antigen versus simple adsorption permits the presentation of natively folded protein for serum antibody capture. Our assay is similar in design to the widely used RBD ELISA first reported by Amanat et al. (6) and later expanded by Stadlbauer et al. (8) In our assay, however, the addition of ACE2-Fc competitor to the sera enables us to test for potentially neutralizing antibodies that block ACE2-RBD interactions. The competition reactions are performed on the same plate and using the same detection protocol to enable rapid, reproducible characterization of a patient’s anti-Spike antibody profile. We found that a high and remarkably consistent proportion of patient antibodies compete with ACE2 for RBD binding.

## RESULTS

### Natively presented SARS-CoV-2 Spike protein antigens effectively detect anti-Spike antibodies.

**FIG 1:**
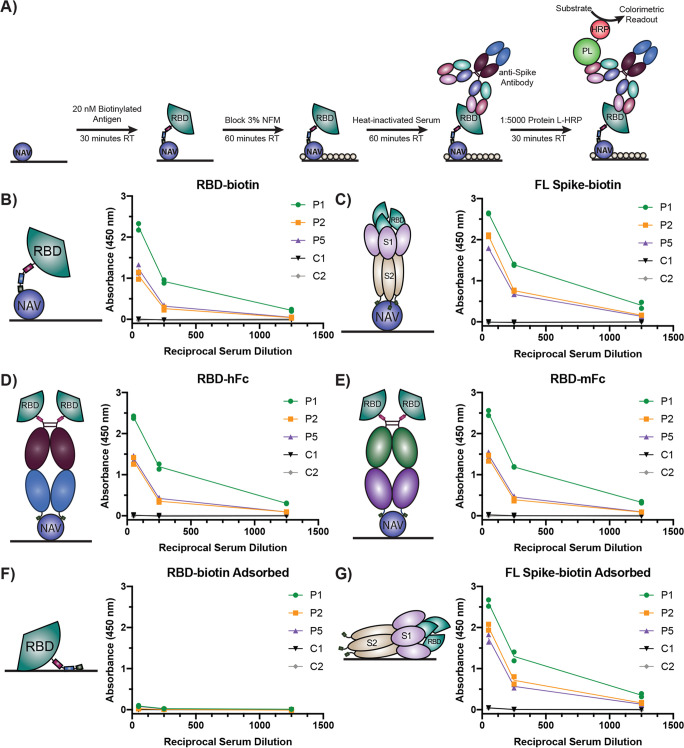
Natively presented SARS-CoV-2 Spike protein antigens effectively detect anti-Spike antibodies. (A) Schematic of NeutrAvidin/biotinylated antigen serology ELISA setup and detection strategy using protein L-HRP (PL-HRP). (B to G) Data represent ELISA results for the indicated antigens presented via NeutrAvidin (NAV, B to E) or passively adsorbed to the plate (F and G). Sera from three patients (P1, P2, and P5) and two healthy controls (C1 and C2) were tested. Antigen coating solutions were 20 nM. Each sample was run with two technical replicates. Dots indicate the mean signal of technical replicates from each of two (*n* = 2) independent experiments. RT, room temperature; NFM, nonfat milk.

Recently, we developed a number of biotinylated SARS-CoV-2 Spike protein antigen and ACE2 formats with broad utility for SARS-CoV-2 research (11). Given current needs for SARS-CoV-2 serological testing, we developed a serological assay using these biotinylated constructs (Fig. 1A; see also Fig. S1 in the supplemental material). Briefly, plates are first coated with NeutrAvidin followed by incubation with biotinylated antigen. Plates are then blocked using 3% nonfat milk and incubated with serum diluted in 1% nonfat milk (Fig. 1A). To test this assay design, our pilot studies utilized sera obtained from an initial cohort of nine patients with a history of positive reverse transcription-PCR test results. Sera in this test cohort were collected at least 14 days following resolution of COVID-19 respiratory and constitutional symptoms. First, as the use of a standard anti-IgG-horseradish peroxidase (HRP) as a detection reagent was precluded by our incorporation of a human Fc region into some antigen constructs for dimeric RBD presentation, we tested multiple alternative detection reagents using a patient from our test cohort. We found that anti-Fab-HRP, anti-IgM-HRP, and protein L-HRP all supported detection of anti-RBD antibodies in patient sera (Fig. S2). Further pilot studies performed with patients from the test cohort revealed that heat inactivation of patient serum (56°C, 60 min) did not significantly reduce signal (*P* = 0.4877, Fig. S3), consistent with previous reports (6), and that coating with RBD-biotin at a concentration as low as 20 nM still provided robust detection of anti-RBD patient antibodies (Fig. S4). In summary, our results converged on optimal assay conditions utilizing a 20 nM antigen coating concentration; 50-fold-diluted, heat-inactivated sera to capture patients across a range of seroreactivity levels; and protein-L-HRP or anti-Fab-HRP as a detection reagent.

10.1128/mSphere.00802-20.1 FIG S1 Protein constructs used in competitive SARS-CoV-2 serology assay. (A) Table of antigen and competitor constructs. (B) SDS-PAGE of proteins used in serology assays. All proteins were prepared as previously described (11) and visualized with SDS-PAGE in either the presence or absence of β-mercaptoethanol and NeutrAvidin (NAV) as indicated. Download FIG S1, JPG file, 0.5 MB. Copyright © 2020 Byrnes et al. 2020 Byrnes et al. This content is distributed under the terms of the Creative Commons Attribution 4.0 International license.

10.1128/mSphere.00802-20.2 FIG S2 HRP-conjugated anti-Fab, anti-IgM, and protein L are effective secondaries. ELISA was performed using the indicated antigens and detection with (A) anti-Fab-HRP, (B) anti-IgM-HRP, or (C) protein L-HRP secondaries for one patient and one healthy control. The buffer condition represents wells that received 1% nonfat milk–PBST instead of serum. Sera were diluted 1:50. Dots represent values determined for technical replicates; bars represent means of results from these replicates. Download FIG S2, JPG file, 0.2 MB. Copyright © 2020 Byrnes et al. 2020 Byrnes et al. This content is distributed under the terms of the Creative Commons Attribution 4.0 International license.

10.1128/mSphere.00802-20.3 FIG S3 Heat inactivation of sera at 56°C for 60 min does not affect detection with protein L-HRP. Data represent ELISA results from wells coated with 20 nM RBD-biotin and incubated with either untreated or heat-inactivated sera from four patients and two healthy controls. Sera were diluted 1:50. Dots represent mean values determined for two technical replicates. Paired *t*-tests were performed to determine statistical significance. Download FIG S3, JPG file, 0.1 MB. Copyright © 2020 Byrnes et al. 2020 Byrnes et al. This content is distributed under the terms of the Creative Commons Attribution 4.0 International license.

10.1128/mSphere.00802-20.4 FIG S4 Effect of antigen coating concentration on protein L-HRP signal. Data represent ELISA results from wells coated with various concentrations of RBD-biotin and incubated with sera from one patient and one healthy control. Sera were diluted 1:50. Download FIG S4, JPG file, 0.1 MB. Copyright © 2020 Byrnes et al. 2020 Byrnes et al. This content is distributed under the terms of the Creative Commons Attribution 4.0 International license.

To profile the efficacy of our various biotinylated antigens for direct detection of anti-Spike antibodies in patient sera, we performed a head-to-head comparison of all antigen constructs listed in Fig. S1A. All of the antigens effectively captured anti-Spike antibodies from three patient sera from the test cohort, whereas sera from two healthy controls were not reactive (Fig. 1B to E). We observed a dose-dependent signal decrease with increasing serum dilution for all antigens, and all three patients tested exhibited strong reactivity to both RBD-biotin and biotinylated full-length (FL) Spike protein ectodomain. Of note, the use of a human Fc fusion (RBD-hFc) or a mouse Fc fusion (RBD-mFc) did not affect signal strength (Fig. 1D and E). Surprisingly, there also did not seem to be a clear benefit of monomeric (RBD-biotin, Fig. 1B) versus dimeric (RBD-hFc, RBD-mFc, Fig. 1D and E) presentation of the RBD, aside from slightly higher signal at a 1:250 serum dilution with the dimeric constructs. This may have been a result of using tetrameric NeutrAvidin to present RBD-biotin, which would mimic an avidity effect.

Interestingly, while passive adsorption of RBD-biotin to the plate instead of utilization of NeutrAvidin resulted in loss of signal (Fig. 1F), adsorption of FL Spike-biotin did not affect signal (Fig. 1G). Adsorption at a higher RBD-biotin concentration (100 nM) yielded signal with protein L-HRP as well as with anti-human IgG in a format analogous to previously reported assays (6, 8) (Fig. S5), indicating that RBD-biotin can also be used in an adsorption format, but at higher concentrations. Not surprisingly, we observed higher signal at all serum dilutions with FL Spike-biotin (413 kDa) than with RBD-biotin (28.5 kDa). However, the signal increase was less than 2-fold, while the size difference between these proteins by molecular weight is >14-fold. This observation suggests that a large proportion of anti-Spike antibodies that patients develop specifically target the RBD and is consistent with findings indicating that Spike glycosylation shields much of the protein’s non-RBD surface from antibody recognition (12). Taken together, these data demonstrate that our biotinylated antigen constructs can be effectively presented using immobilized avidin and offer another option for serologic screening of individuals for anti-SARS-CoV-2 immunity.

10.1128/mSphere.00802-20.5 FIG S5 Comparison of data representing passive adsorption and avidin/biotin presentation of RBD-biotin. RBD-biotin was either adsorbed or bound by NeutrAvidin at a concentration of 20 nM (A and B) or 100 nM (C and D) and detected with protein L-HRP (A and C) or anti-IgG-HRP (B and D). Sera were diluted 1:50. Download FIG S5, JPG file, 0.3 MB. Copyright © 2020 Byrnes et al. 2020 Byrnes et al. This content is distributed under the terms of the Creative Commons Attribution 4.0 International license.

### ACE2-Fc competes with patient antibodies for RBD binding.

**FIG 2:**
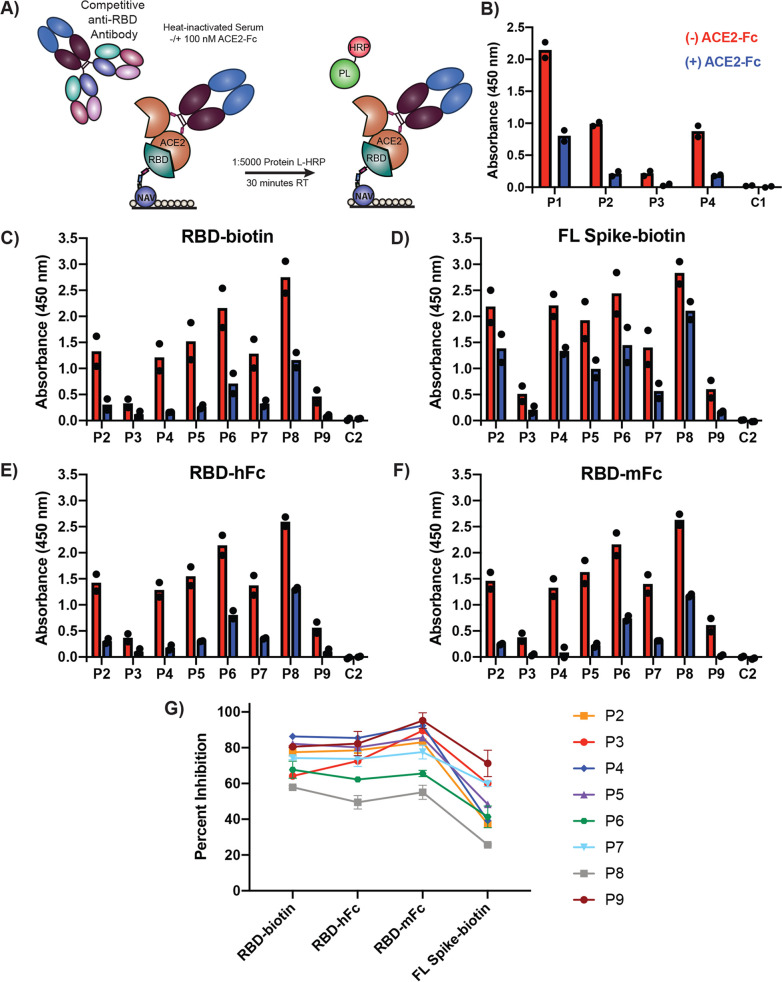
ACE2-Fc competes with patient antibodies for RBD binding. (A) Schematic of ACE2-Fc competitive serology ELISA. (B) Competition ELISA (100 nM ACE2-Fc) results from four patients (P1 to P4) and one healthy control (C1) using RBD-biotin as the capture antigen. (C to F) Competition ELISA results using the indicated antigens for eight patients (P2 to P9) and one healthy control (C2). All sera were diluted 1:50 for analysis, and bound antibodies were detected with protein L-HRP. Each sample was run with two technical replicates. Dots indicate mean signal of technical replicates from two (*n* = 2) independent experiments. Bars show the means of results from these two experiments. (G) Percent inhibition of signal seen with competition. Dots represent means ± SD (*n* = 2).

We next adapted our assay to incorporate a competition condition where patient antibodies compete with ACE2 to bind Spike antigen on the ELISA plate (Fig. 2A). This design represents a straightforward means to assess the global capacity of a patient’s serum antibodies to compete with ACE2 for RBD binding. We first tested monomeric ACE2 and observed a modest but consistent reduction in bound antibody signal across four patient samples from our test cohort (Fig. S6). We have previously shown dimeric ACE2-Fc binds ∼4-fold more tightly to monomeric RBD (11). Therefore, we postulated that the improved affinity and potential avidity afforded with this dimeric construct would allow greater competition with patient antibodies. Indeed, we observed a much greater decrease in RBD binding of patient antibodies when serum was supplemented with ACE2-Fc at 100 nM (Fig. 2B), a concentration of ACE2-Fc that we found to cause saturation of RBD on the plate (Fig. S7). Pretreating the antigen-coated plate with ACE2-Fc prior to adding serum produced slightly higher signal than adding ACE2-Fc to serum. This suggests that ACE2-Fc pretreatment allows some dissociation of ACE2-Fc during serum incubation and, consequently, increased patient antibody binding (Fig. S8). Therefore, we chose to supplement sera with ACE2-Fc to allow simultaneous competition with the patient antibodies.

10.1128/mSphere.00802-20.6 FIG S6 ACE2 monomer competes with patient anti-RBD antibodies only minimally. (A) Schematic of monomeric ACE2 competitive serology ELISA setup. (B) ELISA results from wells coated with 20 nM RBD-biotin and incubated with sera ± 100 nM monomeric ACE2 from four patients (P1 to P4) and two healthy controls (C1 and C2). Sera were diluted 1:50. Dots represent values determined for technical replicates; bars represent means of results from these replicates. Download FIG S6, JPG file, 0.2 MB. Copyright © 2020 Byrnes et al. 2020 Byrnes et al. This content is distributed under the terms of the Creative Commons Attribution 4.0 International license.

10.1128/mSphere.00802-20.7 FIG S7 ACE2-Fc saturates plate-bound RBD-biotin and FL Spike-biotin at 100 nM. ACE2-Fc binding was determined using a protocol analogous to the serology ELISA protocol. Instead of serum, 3-fold serial dilutions of ACE2-Fc in 1% nonfat milk were incubated in wells coated with NeutrAvidin-presented RBD-biotin or FL Spike-biotin (20 nM coating concentration). ACE2-Fc binding was detected using anti-IgG-HRP. Data are from one experiment. Download FIG S7, JPG file, 0.1 MB. Copyright © 2020 Byrnes et al. 2020 Byrnes et al. This content is distributed under the terms of the Creative Commons Attribution 4.0 International license.

10.1128/mSphere.00802-20.8 FIG S8 Pretreatment with ACE2-Fc and addition of ACE2-Fc to serum yield similar competition profiles. Data represent ELISA results from wells coated with 20 nM solutions of the indicated antigens and incubated with sera from four patients and one healthy control. Sera were added with no ACE2-Fc (red), were added to wells pretreated with 100 nM ACE2-Fc and then washed before serum addition (orange), or were supplemented with 100 nM ACE2-Fc during incubation in the well (blue). Sera were diluted 1:50. Dots represent values determined for technical replicates; bars represent means of results from these replicates. Download FIG S8, JPG file, 0.4 MB. Copyright © 2020 Byrnes et al. 2020 Byrnes et al. This content is distributed under the terms of the Creative Commons Attribution 4.0 International license.

To determine the patient-to-patient variability in our ACE2-Fc competition serology assay, and to test our various antigen formats in this competition mode, we expanded our efforts to test additional convalescent patients in our test cohort as well as a healthy control. We observed a 10-fold range of variation in the overall anti-Spike signal between patients, and the trends were consistent between antigens (Fig. 2C to F). Specifically examining the antigens containing RBD alone (RBD-biotin, RBD-hFc, and RBD-mFc, Fig. 2C, E, and F), all patients exhibited differing but substantial degrees of signal decrease when ACE2-Fc was added to the serum (50% to 95%, Fig. 2G). This finding suggests that the patients in this small cohort had all generated anti-RBD antibodies that bind at or near the ACE2-binding RBD epitope.

Interestingly, when FL Spike-biotin was used as the antigen (Fig. 2D), the signals for both direct detection and ACE2-Fc competition were largely elevated relative to those seen with the antigens containing RBD alone. The average percentage of inhibition of signal with ACE2-Fc competition was also lower than that seen with the antigens containing RBD alone (Fig. 2G). These observations likely represent anti-Spike antibodies that bind outside the RBD that are unaffected by ACE2-Fc competition. Taken together, these results demonstrate the utility of our ACE2-Fc competition assay for simultaneously determining both baseline serum reactivity to Spike antigens and whether a serum sample contains antibodies that can block ACE2 binding.

### Patients produce a consistent proportion of competitive anti-RBD antibodies.

**FIG 3:**
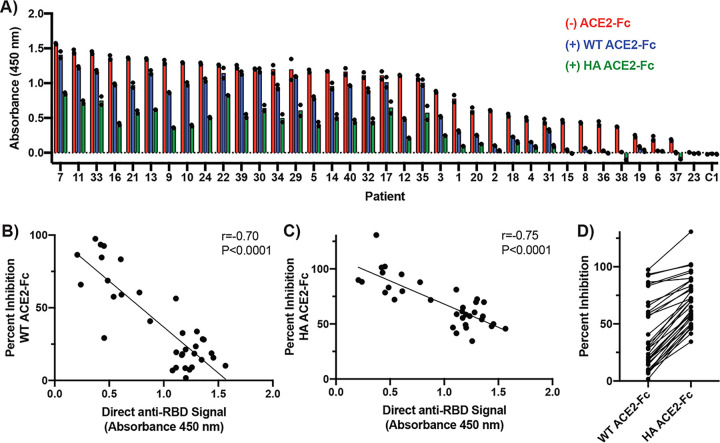
A high-affinity ACE2-Fc variant enhances competition with patient antibodies. (A) Competition ELISA results from 36 patients obtained using a 100 nM concentration of either wild-type (WT) or high-affinity (HA) ACE2-Fc and RBD-biotin as the capture antigen. All sera were diluted 1:50 for analysis, and bound antibodies were detected with anti-Fab-HRP. Each sample was run once with two technical replicates. Dots indicate signal of each technical replicate. Bars show the means of results from these two replicates. (B and C) Correlation of direct anti-RBD signal and percent signal inhibition with competition using either WT ACE2-Fc (B) or HA ACE2-Fc (C). Patients with direct anti-RBD signal values of <0.2 were excluded from percent decrease analysis (2/36). (D) Compiled percent inhibition data for each ACE2-Fc variant. Lines connect values representing results from the same patient.

Given the success of the competition assay in testing our small pilot cohort, we next tested a cohort of 36 sera from PCR-positive patients using RBD-biotin as the capture antigen (Fig. 3A). This expanded cohort revealed a much larger range in the percentages of inhibition of RBD-biotin-binding signal with ACE2-Fc than was seen with our pilot cohort (2% to 97% versus 58% to 86%, Fig. 3B). Interestingly, there was a strong negative correlation between the direct anti-RBD signal and the percentage of inhibition with ACE2-Fc (r = −0.70, *P* < 0.0001, Fig. 3B), suggesting that patients with high levels of anti-RBD antibodies either (i) produce a higher proportion of noncompetitive antibodies or (ii) produce antibodies with higher affinities or concentrations that are capable of outcompeting ACE2-Fc for RBD binding. To determine if these high-signal patients had antibodies of sufficient concentration or affinity to outcompete ACE2-Fc in our assay, we performed the competition assay using a higher-affinity ACE2-Fc variant (high-affinity [HA] ACE2-Fc) that we recently developed using Rosetta design and yeast display (5). This variant exhibited ∼39-fold-higher RBD-binding affinity than wild-type ACE2-Fc. Notably, at the same 100 nM concentration, HA ACE2-Fc yielded much higher reductions in signal, especially in individuals with high anti-RBD seropositivity (34% to 131%, Fig. 3A, C, and D). This finding suggests that in the competition assay, patients with high anti-RBD signal likely have either higher-affinity antibody clones or sufficiently high concentrations of anti-RBD antibodies to outcompete even 100 nM wild-type ACE2-Fc. Therefore, in subsequent experiments, we utilized HA ACE2-Fc as the competitor to ensure that we could detect the presence of competitive antibodies in highly seropositive patients.

**FIG 4:**
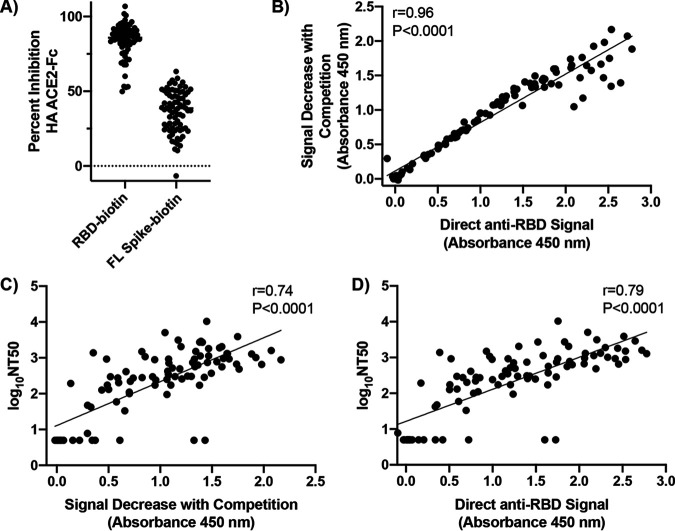
Patients produced a consistent proportion of competitive anti-RBD antibodies. (A) Compiled percent signal inhibition with RBD-biotin or FL Spike-biotin as the capture antigen in competition assay using 100 nM HA ACE2-Fc. All sera were diluted 1:50 for analysis, and bound antibodies were detected with anti-Fab-HRP. Each sample was run once with two technical replicates. Dots represent mean values obtained from these two replicates. Patients with direct anti-RBD signal values of <0.2 were excluded from percent inhibition analysis (22/99). (B) Correlation of direct anti-RBD signal and signal decrease with HA ACE2-Fc competition. (C and D) Correlation of signal decrease with either HA ACE2-Fc (C) or direct anti-RBD signal (D) with NT50 values published for these patients by Robbiani et al. (13).

As a final test of our competitive assay, we analyzed 99 convalescent-phase sera from a cohort previously published with corresponding pseudovirus neutralization data (13). This cohort was comprised predominantly of outpatients and included only two hospitalized individuals. The average duration of symptoms was 10.4 ± 5.6 days, and samples were collected an average of 34.0 ± 8.2 days after symptom onset (13). With this cohort, we again observed a strikingly consistent proportion of competitive anti-RBD antibodies in patient sera with a direct anti-RBD signal of <0.2 in our assay (83 ± 11%, 50% to 107% signal inhibition, Fig. 4A). As with our early pilot cohorts, the use of FL Spike as the capture antigen led to lower signal inhibition (37% ± 14%, −7% to 63%, Fig. 4A), again likely as a result of anti-Spike antibodies binding outside the RBD that are not competitive with HA ACE2-Fc. Of note, an expanded group of 27 healthy control sera included in these experiments did not show any seropositivity or competition (Fig. S9). In examining the competition data for this cohort utilizing RBD-biotin as the capture antigen, a strong positive correlation (*r* = 0.96, *P* < 0.0001) was observed between direct anti-RBD signal and the magnitude of signal lost with HA ACE2-Fc competition for all 99 patients, underscoring the consistent proportion of competitive antibodies produced in these patients (Fig. 4B). Here, we again observed a few high-signal patients deviating from the tight correlation, suggesting that these individuals may have had sufficiently high concentrations of competitive antibodies, or competitive antibodies of sufficient affinity, to compete with even 100 nM HA ACE2-Fc. Similar trends were also observed with FL Spike as the capture antigen, but the correlation was weaker given mixed detection of both anti-RBD and anti-Spike antibodies (Fig. S10).

10.1128/mSphere.00802-20.9 FIG S9 Control sera obtained before the outbreak of SARS-CoV-2 are not seropositive for anti-RBD or anti-Spike antibodies. Data represent compiled results from competition assay using RBD-biotin (A) or FL Spike-biotin (B) as the capture antigen with 100 nM HA ACE2-Fc as the competitor. All sera were diluted 1:50 for analysis, and bound antibodies were detected with anti-Fab-HRP. Each sample was run once with two technical replicates on the plate. Bars represent means of results from these two technical replicates. Download FIG S9, JPG file, 0.3 MB. Copyright © 2020 Byrnes et al. 2020 Byrnes et al. This content is distributed under the terms of the Creative Commons Attribution 4.0 International license.

10.1128/mSphere.00802-20.10 FIG S10 Results of competition assay for cohort of 99 patients performed using FL Spike-biotin as the capture antigen. FL Spike-biotin was used as the capture antigen in competition assay with 100 nM HA ACE2-Fc as the competitor. All sera were diluted 1:50 for analysis, and bound antibodies were detected with anti-Fab-HRP. Each sample was run once with two technical replicates on the plate. Dots represent means of results from these two replicates. (A) Correlation of direct anti-FL Spike signal and raw signal inhibition with competition. (B and C) Correlation of either raw signal inhibition (B) or direct anti-FL Spike signal (C) with NT50 values published previously for these patients by Robbiani et al. (13). Download FIG S10, JPG file, 0.3 MB. Copyright © 2020 Byrnes et al. 2020 Byrnes et al. This content is distributed under the terms of the Creative Commons Attribution 4.0 International license.

Finally, we compared the results from our competition assay with published pseudovirus neutralization data generated for these patients (13). Half-maximal neutralizing titer (NT50) showed a positive and significant correlation (*r* = 0.74, *P* < 0.0001, Fig. 4C) with raw signal inhibition, suggesting that there may be predictive value of our competition assay with respect to neutralization potential of patient sera. Assessing the relationship between direct anti-RBD signal in our assay and NT50, we observed a similar positive correlation (*r* = 0.79, *P* < 0.0001, Fig. 4D), consistent with the initial publication characterizing these samples (13). Therefore, given the highly consistent proportion of competitive antibodies in patients, the competition mode of our assay provides resolution similar to that of direct RBD seropositivity in terms of predicting serum neutralizing activity.

## DISCUSSION

As the SARS-CoV-2 pandemic escalates, there is a continued need for assays to profile patient responses to infection, especially with respect to the antiviral antibodies generated and whether or not a patient has acquired humoral immunity against SARS-CoV-2. An important advance presented here is the implementation of a straightforward means to assess the global capacity of a patient’s serum antibodies to compete with ACE2 for RBD binding. By simply adding ACE2-Fc to the serum dilution buffer, we modified our direct-detection ELISA to reveal the presence of antibodies that bind at a potentially neutralizing RBD epitope in the ACE2/RBD interface. We found that essentially all anti-RBD seropositive patients tested had antibodies that bound the RBD at or near this interface, as indicated by reductions in signal strength in the competition mode of our ELISA, and that the anti-RBD signal strongly correlated with neutralizing activity. These findings not only indicate that the ACE2-binding surface of the RBD is highly immunogenic but also suggest that most COVID-19 patients develop antibodies against this potentially neutralizing epitope. In the context of previous findings indicating that SARS-CoV neutralizing antibodies bind the Spike RBD and block ACE2 binding (14, 15), our data suggest that this premise is also true for SARS-CoV-2. Furthermore, our results indicate that in most of the patients tested here, a majority of the anti-RBD antibodies bound at the ACE2 binding site on the RBD. Collectively, these observations suggest that vaccine development efforts should aim to elicit the generation of these competitive antibodies. However, recent studies have revealed that T cell-mediated immunity may also play an important role in combating SARS-CoV-2 (16). Therefore, the ideal vaccine will likely stimulate both the production of neutralizing antibodies and the development of a memory T cell response. Our assay thus represents a valuable tool to monitor the development of competitive antibodies postvaccination and to support such vaccine design campaigns.

To our knowledge, only two other SARS-CoV-2 studies have examined the ability of serum antibodies to compete with ACE2 for RBD binding (17, 18). Interestingly, in contrast to our findings, one of those studies found that only 3 of 26 patients tested positive for ACE2-competitive antibodies (17). These divergent results possibly represent a consequence of differing assay designs, differing competitor affinities and concentrations, or differing criteria for selection of patient cohorts. However, the second study used an assay orientation to detect competitive patient antibodies that differed from ours but resulted in the observation that a high proportion of the patients had competitive antibodies, consistent with our findings (18). Of note, we found in our experiments that ACE2 monomer could not efficiently compete with patient antibodies for binding, which underscores the importance of our use of a strong, bivalent binder to block the Spike-patient antibody interaction in such a competitive serology assay. Furthermore, at the serum dilution used, we found that the use of a high-affinity ACE2-Fc variant was required to detect competitive antibodies in patients with high anti-RBD signal. Lastly, our assay format could be easily adapted to evaluate if epitopes targeted by new anti-RBD therapies are similar to epitopes targeted by patient antibodies.

In summary, we designed and employed an assay to identify potentially neutralizing antibodies in convalescent patient sera that bind at the ACE2/Spike RBD interface. Using a variety of biotinylated Spike antigens and presentation of natively folded protein via avidin-biotin interactions, we developed an ELISA format for directly measuring patient seroreactivity to the SARS-CoV-2 Spike protein. Competition with ACE2-Fc clearly revealed the presence of potential neutralizing antibodies that bound the RBD in most patients tested and that these antibodies made up a majority of the anti-RBD antibody pool in COVID-19 patients. This new assay represents a high-throughput and simple means of providing additional resolution of the patient antibody response to SARS-CoV-2 infection, and the consistent proportion of patient antibodies that competed with ACE2 for RBD binding further justifies efforts to design therapies and vaccines that block this interaction.

## MATERIALS AND METHODS

### Antigen generation.

All antigens and ACE2 constructs were produced as previously described (11). RBD-mFc was generated by subcloning the RBD DNA sequence into a vector containing a C-terminal mIgG2a-Fc with an Avi tag. The high-affinity ACE2-Fc variant was developed using combined Rosetta design and yeast display (5). The sequence maps for all plasmids are available upon request. Briefly, proteins were expressed in Expi293 cells coexpressing BirA using an ExpiFectamine expression system kit in accordance with the recommended protocol of the manufacturer (Thermo Fisher Scientific). Biotinylated proteins were then purified using either nickel-nitrilotriacetic acid (Ni-NTA) chromatography (RBD-biotin, FL Spike-biotin) or protein A chromatography (RBD-hFc, RBD-mFc) and subjected to buffer exchange into phosphate-buffered saline (PBS) for storage at –80°C. Protein purity was assessed using SDS-PAGE. Biotinylation was confirmed by NeutrAvidin (Thermo Fisher Scientific) shift assay.

### Patient serum.

All serum samples were obtained using protocols approved by the Institutional Review Boards (IRB) of the University of California, San Francisco (UCSF); Stanford University; and Rockefeller University and in accordance with the Declaration of Helsinki. All participants provided written consent.

Blood samples from patients in the pilot cohort (Fig. 1; see also Fig. 2) were obtained via antecubital venipuncture and collected into BD Vacutainer serum collection tubes (UCSF IRB Protocol 20-30338). All patients in this pilot cohort had a positive clinical nasopharyngeal reverse transcription-PCR test result to document SARS-CoV-2 infection. At the time of their blood draw, more than 14 days had elapsed since their COVID-19 respiratory and constitutional symptoms had resolved. Deidentified serum was aliquoted, flash frozen in liquid nitrogen, and stored at –80°C in single-use aliquots. Control samples were obtained from healthy individuals before the emergence of SARS-CoV-2. The 36 patient sera included in the PCR-positive cohort (Fig. 3, remnant sera obtained from Kaiser Permanente of Northern California via Stanford University IRB Protocol 55718) were provided as deidentified, heat-inactivated, neat serum aliquots and were stored at –80°C. The previously published 99 patient sera (Fig. 4, a kind gift of Michel Nussenzweig, Marina Caskey, and Christian Gaebler of Rockefeller University, collected with Rockefeller IRB protocol DRO-1006) (13) and additional control samples (see Fig. S9 in the supplemental material) were provided as deidentified aliquots diluted 1:1 in a reaction mixture containing 40% glycerol, 40 mM HEPES (pH 7.3), 0.04% NaN_3_, and PBS and stored at 4°C. Heat inactivation of all sera was performed by incubating the samples at 56°C for 60 min.

### Competition ELISA protocol.

All assays were performed in 384-well Nunc MaxiSorp flat-bottom plates (Thermo Fisher Scientific), and each sample was run in duplicate. First, plates were coated with 50 μl of 0.5 μg/ml NeutrAvidin or 20 μl of 20 nM antigen (for passively adsorbed antigens) mixed in PBS for 60 min at room temperature. For assays using 100 nM biotinylated antigen, 10 μg/ml NeutrAvidin was used. Plates were then washed three times with PBS containing 0.05% Tween 20 (PBST) and were washed similarly for each of the following steps. Next, 20 μl of biotinylated antigens was added to NeutrAvidin-coated wells and allowed to bind for 30 min at room temperature. After washing, plates were then blocked for 60 min with 80 μl 3% nonfat milk (Lab Scientific)–PBST–10 μM biotin. Sera were diluted in 1% nonfat milk–PBST as indicated in the absence (direct detection) or presence (competition) of 100 nM ACE2-Fc, and 20-μl volumes of these dilutions were incubated in the plates for 60 min at room temperature. The plates were again washed, and antibodies bound to the coated antigens were detected using 20 μl of anti-human Fab (Jackson ImmunoResearch Laboratories 109-036-097 [1:5,000]), anti-human IgM (Sigma-Aldrich A6907 [1:3,000]), anti-human IgG (Sigma-Aldrich A0170 [1:3,000]), or protein L (Thermo Fisher Scientific 32420 [1:5,000]) as indicated for 30 min at room temperature. All detection reagents were conjugated to HRP. Following a final wash, plates were developed for 3 to 10 min at room temperature using 20 μl of 50/50 3,3′,5,5′-tetramethylbenzidine (TMB)/solution B (VWR International). Reactions were quenched with 20 μl 1 M phosphoric acid, and absorbance was measured at 450 nm using a Tecan Infinite M200 Pro spectrophotometer.

### Data analysis and statistics.

Background from the raw ELISA signal was removed by first subtracting the signal measured in wells coated with NeutrAvidin alone or empty wells (passively adsorbed antigens). Next, the signal measured in antigen-coated wells incubated with 1% nonfat milk (direct detection) or 1% nonfat milk–100 nM ACE2-Fc (competition) was subtracted from the signal in serum-treated wells. As there is some detectable reactivity of protein L-HRP to Fc-containing antigens (RBD-hFc, RBD-mFc) and RBD-bound ACE2-Fc (competition mode), this buffer subtraction step is necessary with that secondary. For experiments where samples from the same cohort were spread across multiple plates, a common control was included on all plates for plate-to-plate signal normalization. All graphing and statistical analysis was performed in GraphPad Prism (Version 8.4.2). For the heat treatment comparison, paired *t* tests were used. Where indicated, Spearman’s correlation coefficients were determined and a two-tailed *P* value reported. *P* values of <0.05 were considered statistically significant.
