# Supplementary figures and images for: Competitive SARS-CoV-2 Serology Reveals Most Antibodies Targeting the Spike Receptor-Binding Domain Compete for ACE2 Binding
Source: mSphere. 2020 Sep 16;5(5):e00802-20. doi: 10.1128/mSphere.00802-20 (PMC7494835; doi:10.1128/mSphere.00802-20)

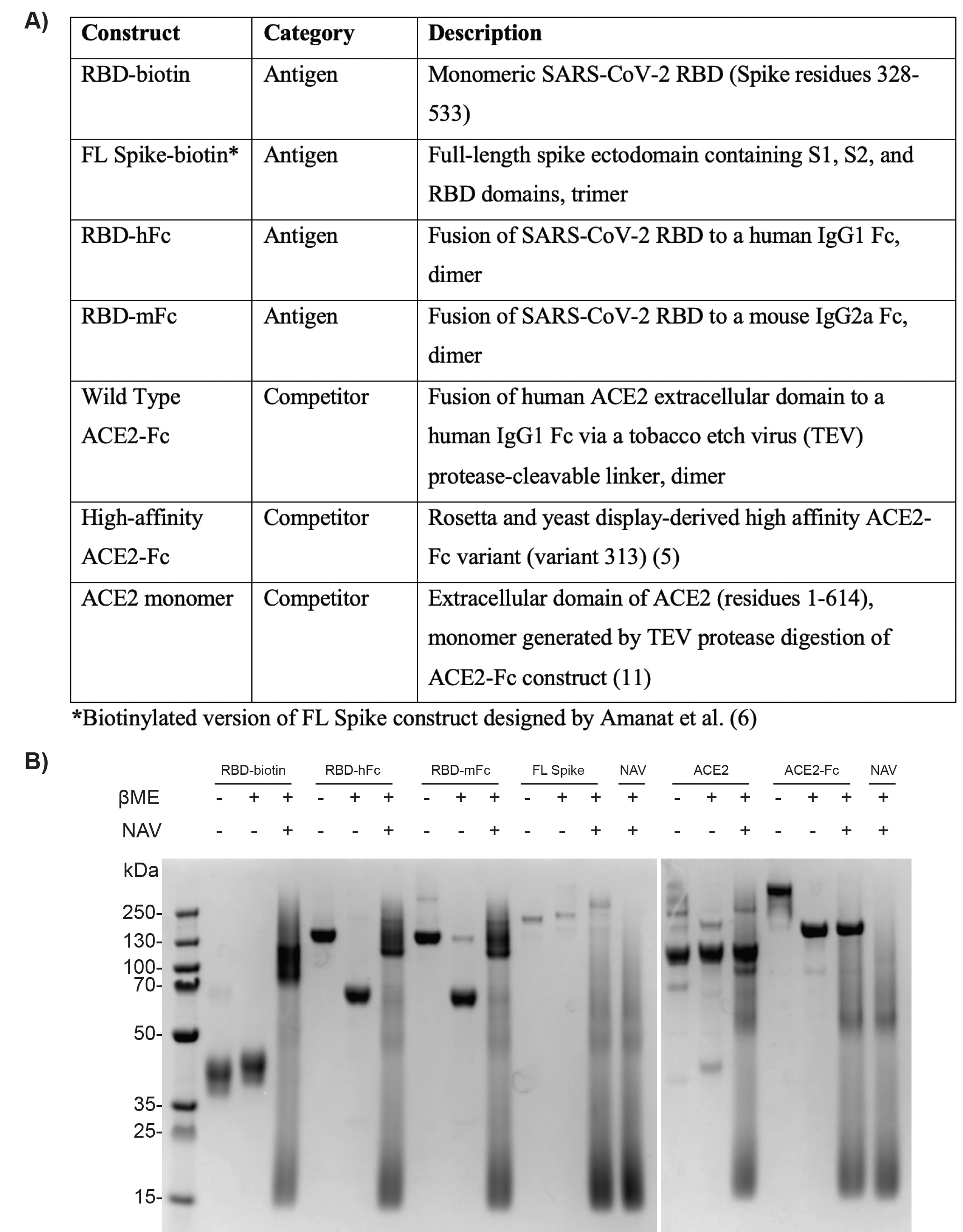

Supplement: FIG S1 [file mSphere.00802-20-sf001.jpg]

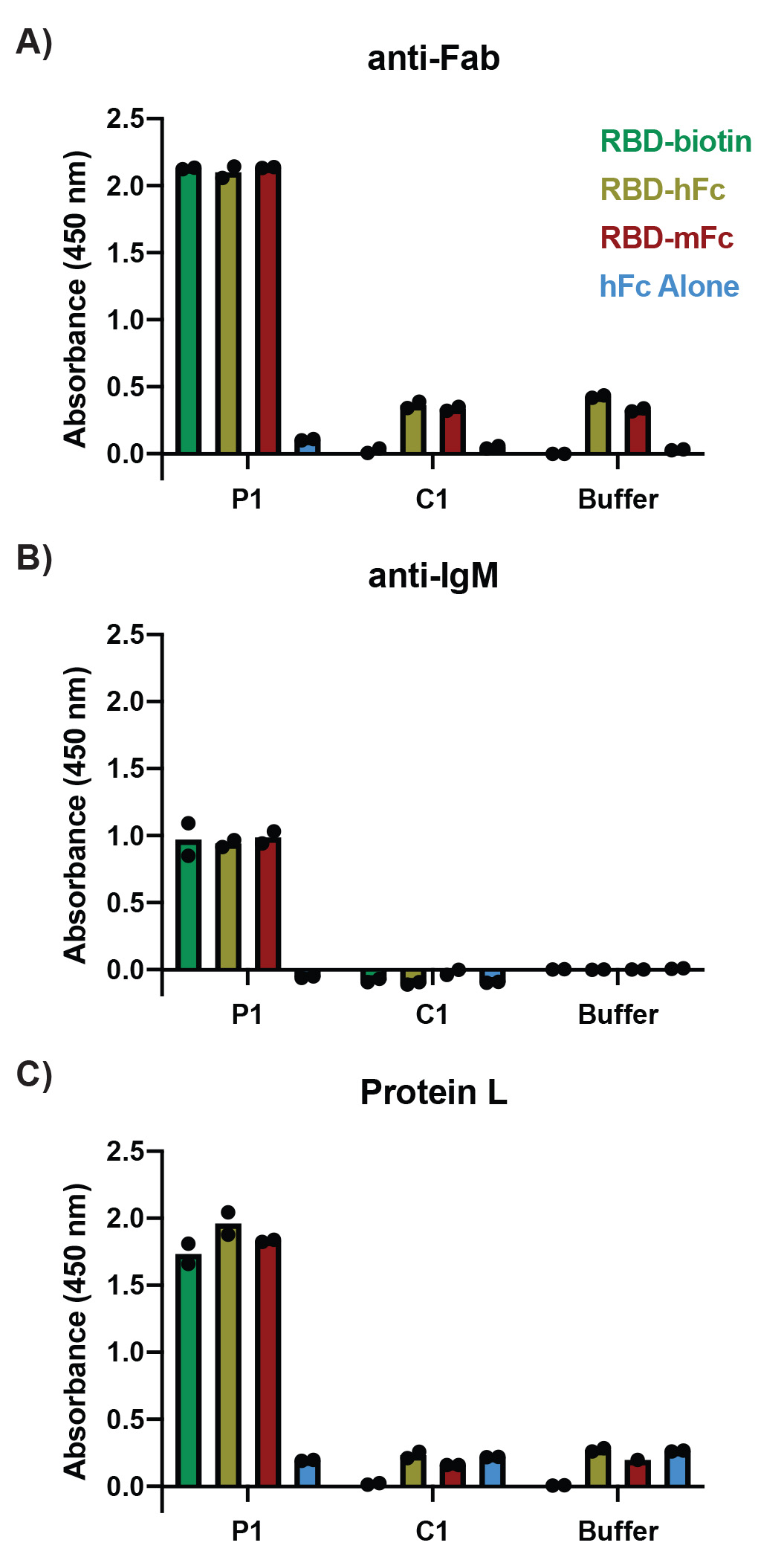

Supplement: FIG S2 [file mSphere.00802-20-sf002.jpg]

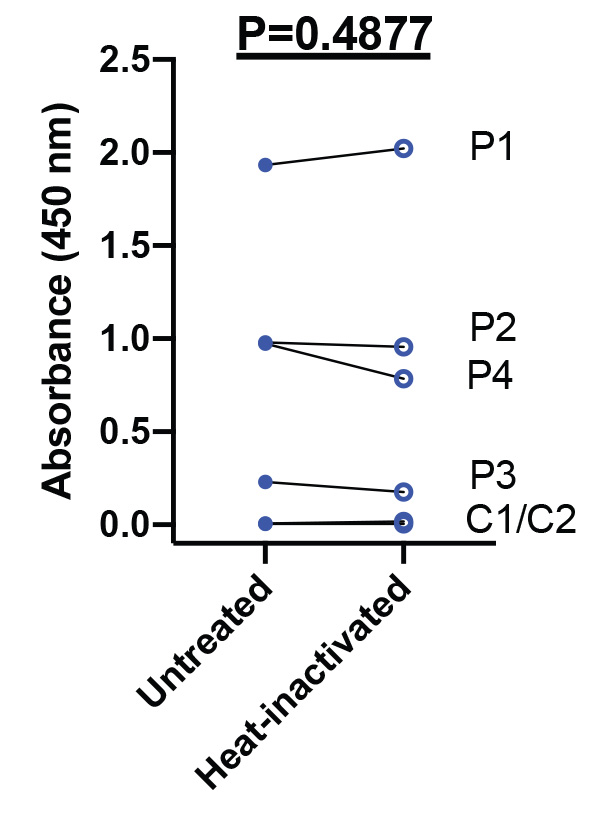

Supplement: FIG S3 [file mSphere.00802-20-sf003.jpg]

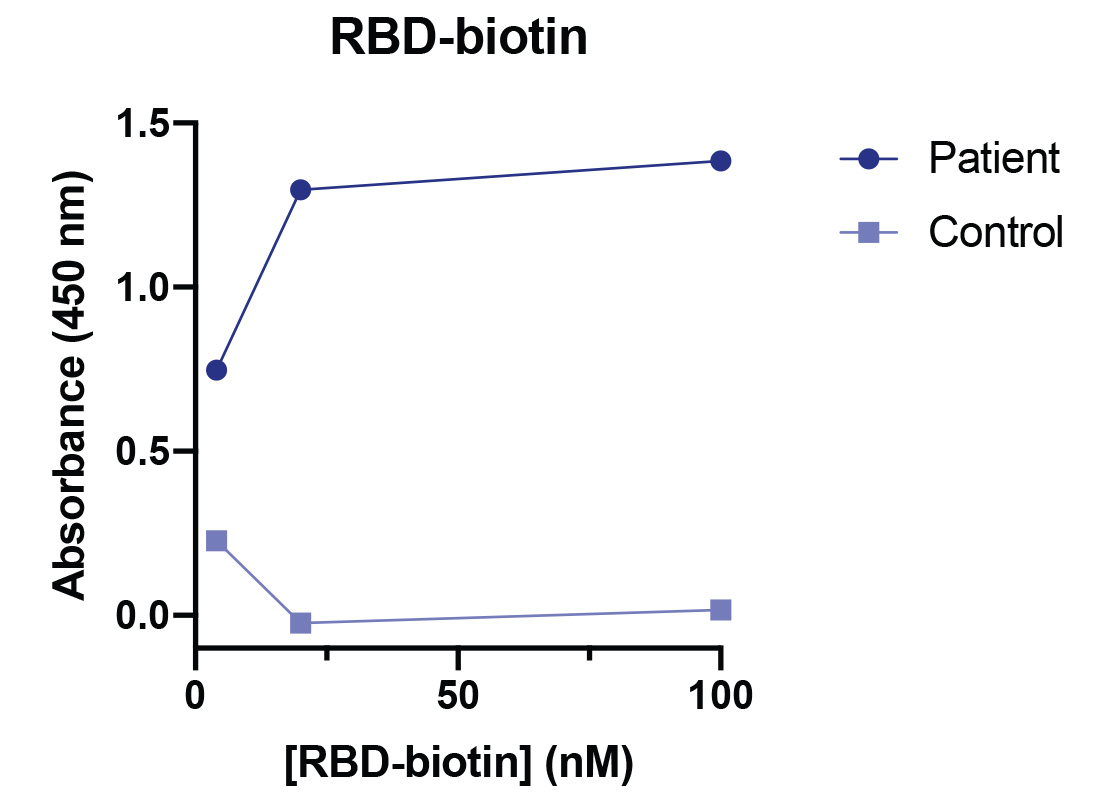

Supplement: FIG S4 [file mSphere.00802-20-sf004.jpg]

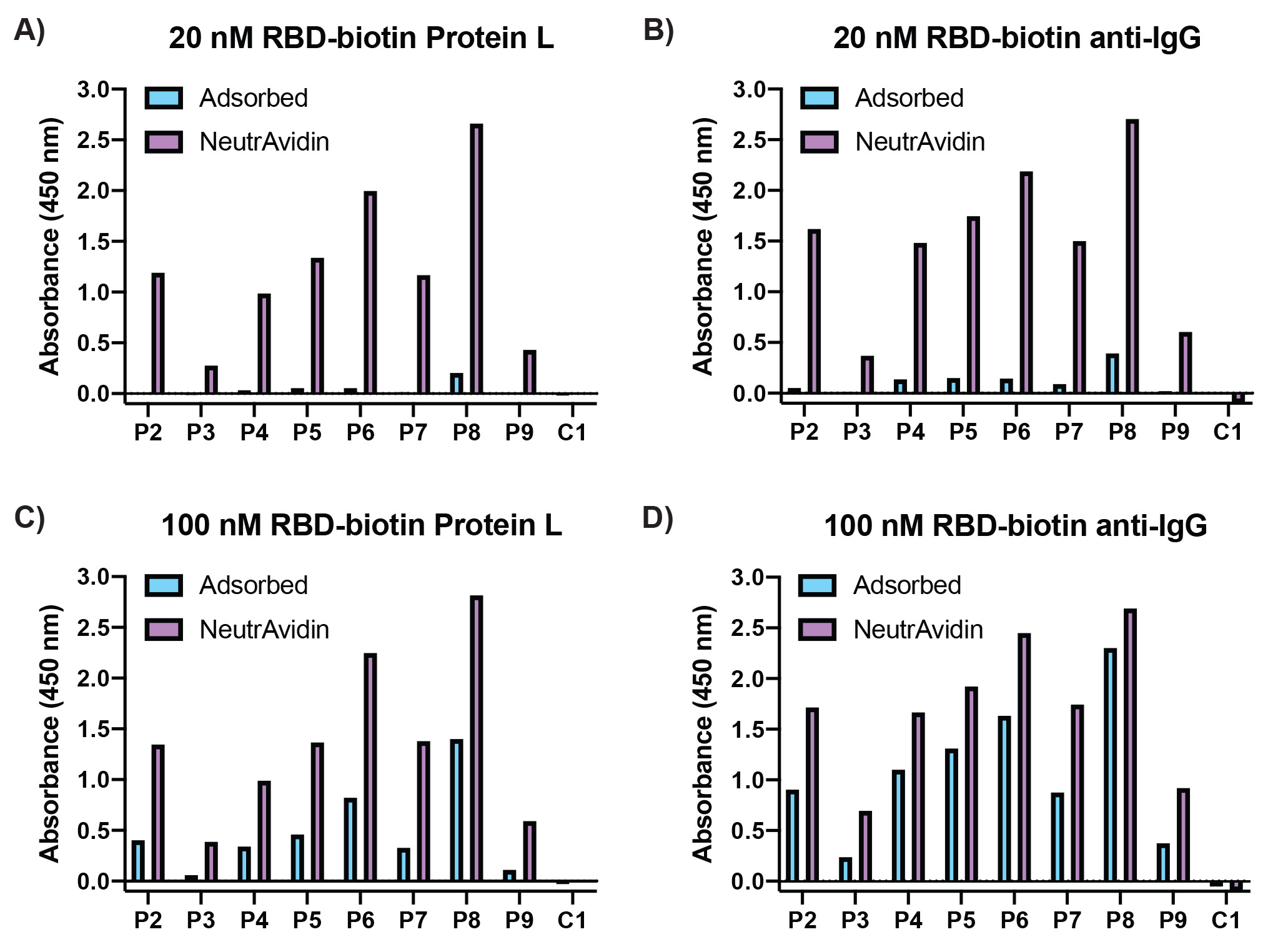

Supplement: FIG S5 [file mSphere.00802-20-sf005.jpg]

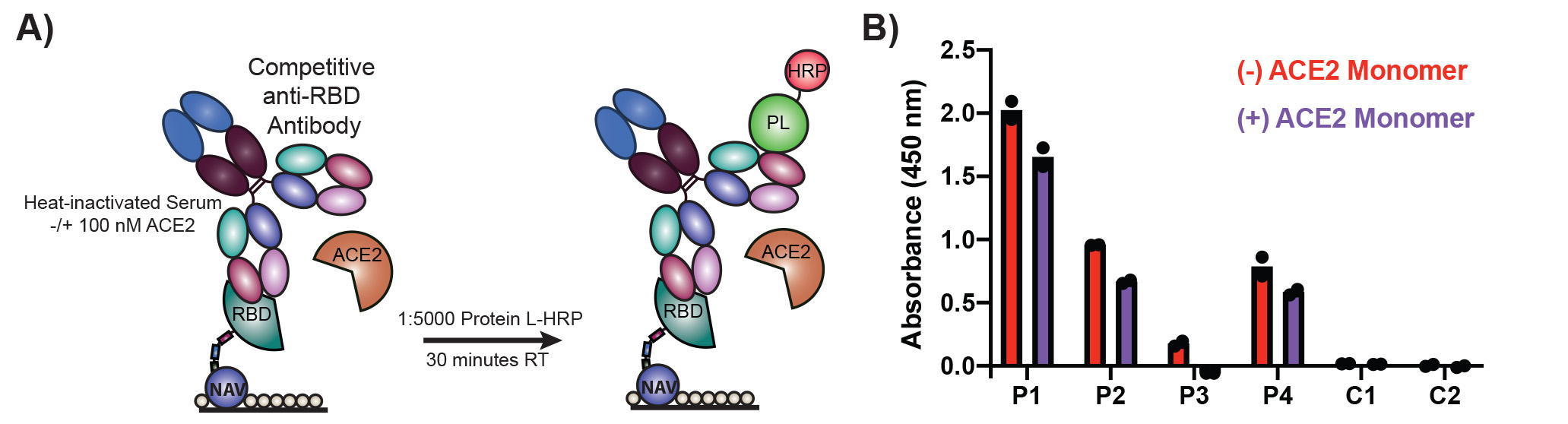

Supplement: FIG S6 [file mSphere.00802-20-sf006.jpg]

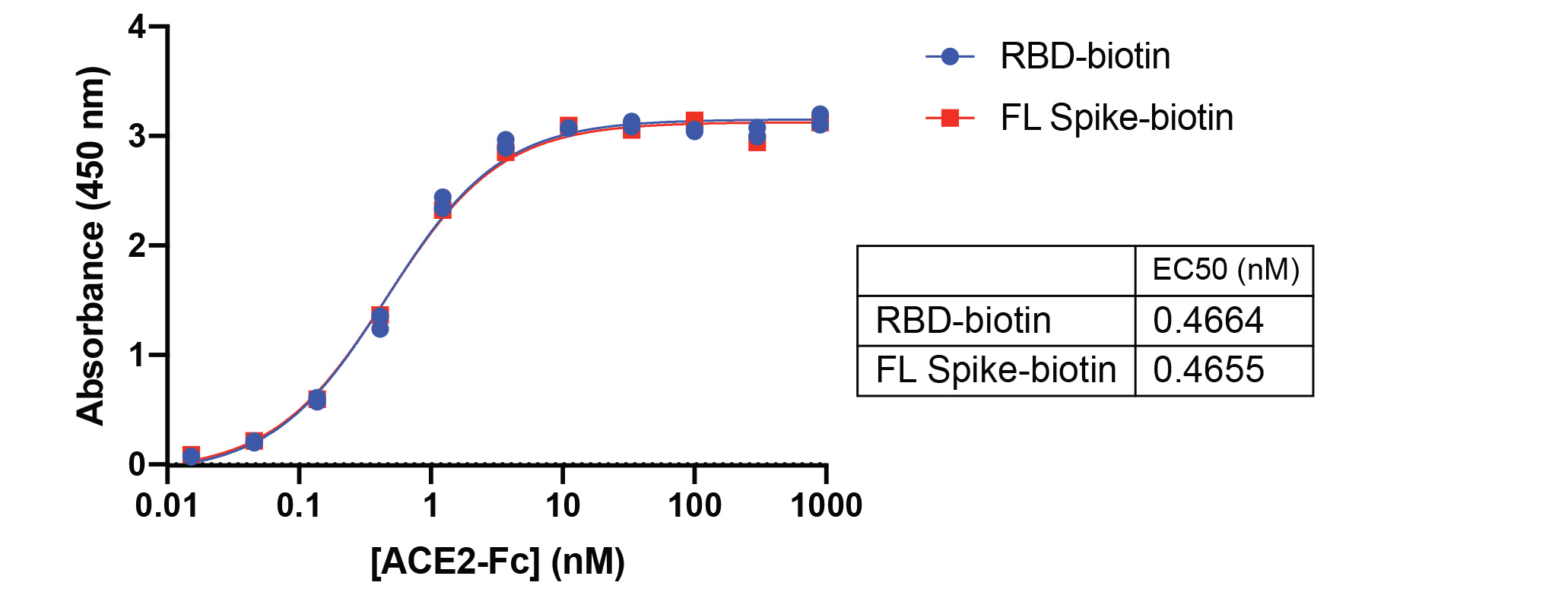

Supplement: FIG S7 [file mSphere.00802-20-sf007.jpg]

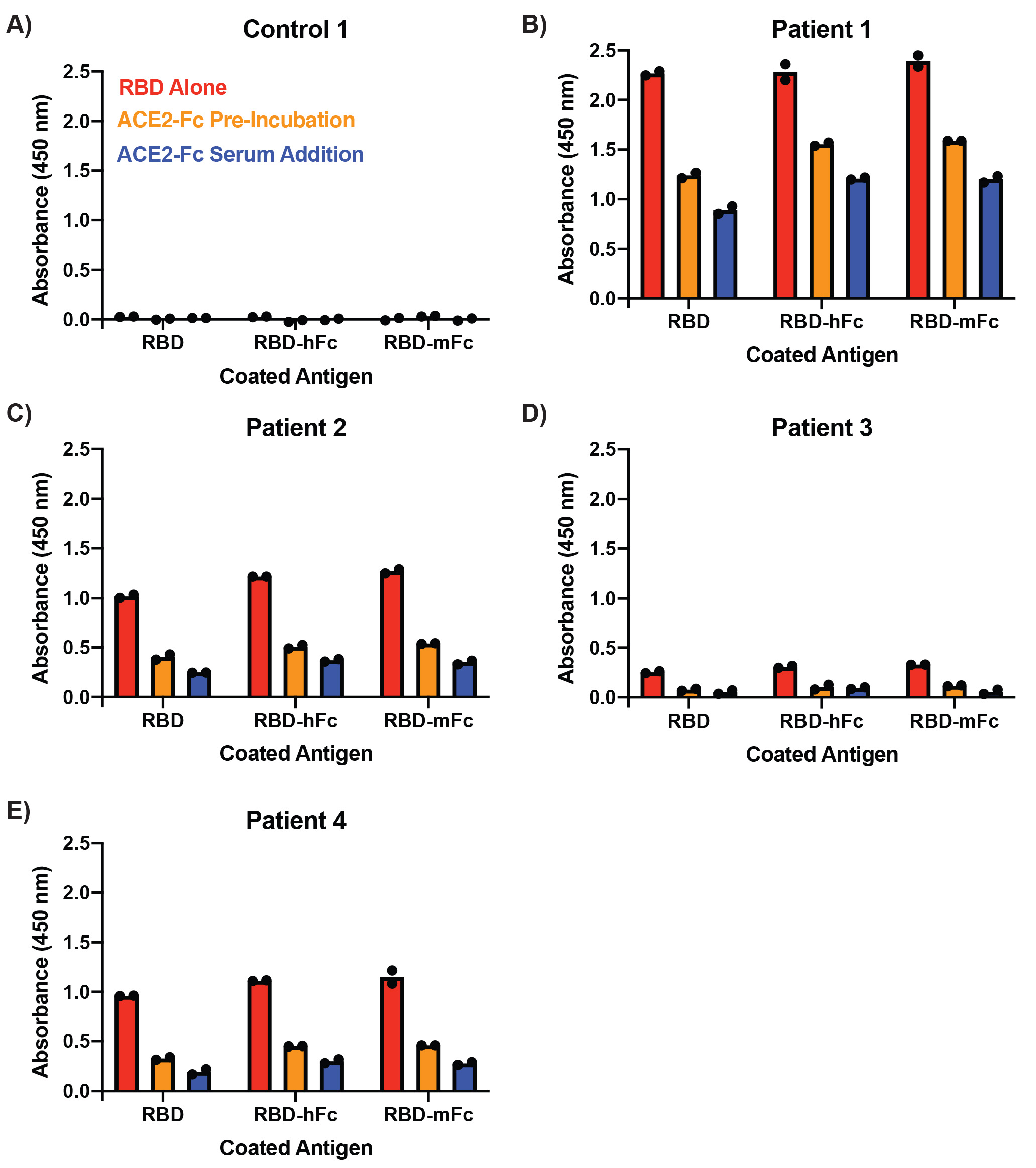

Supplement: FIG S8 [file mSphere.00802-20-sf008.jpg]

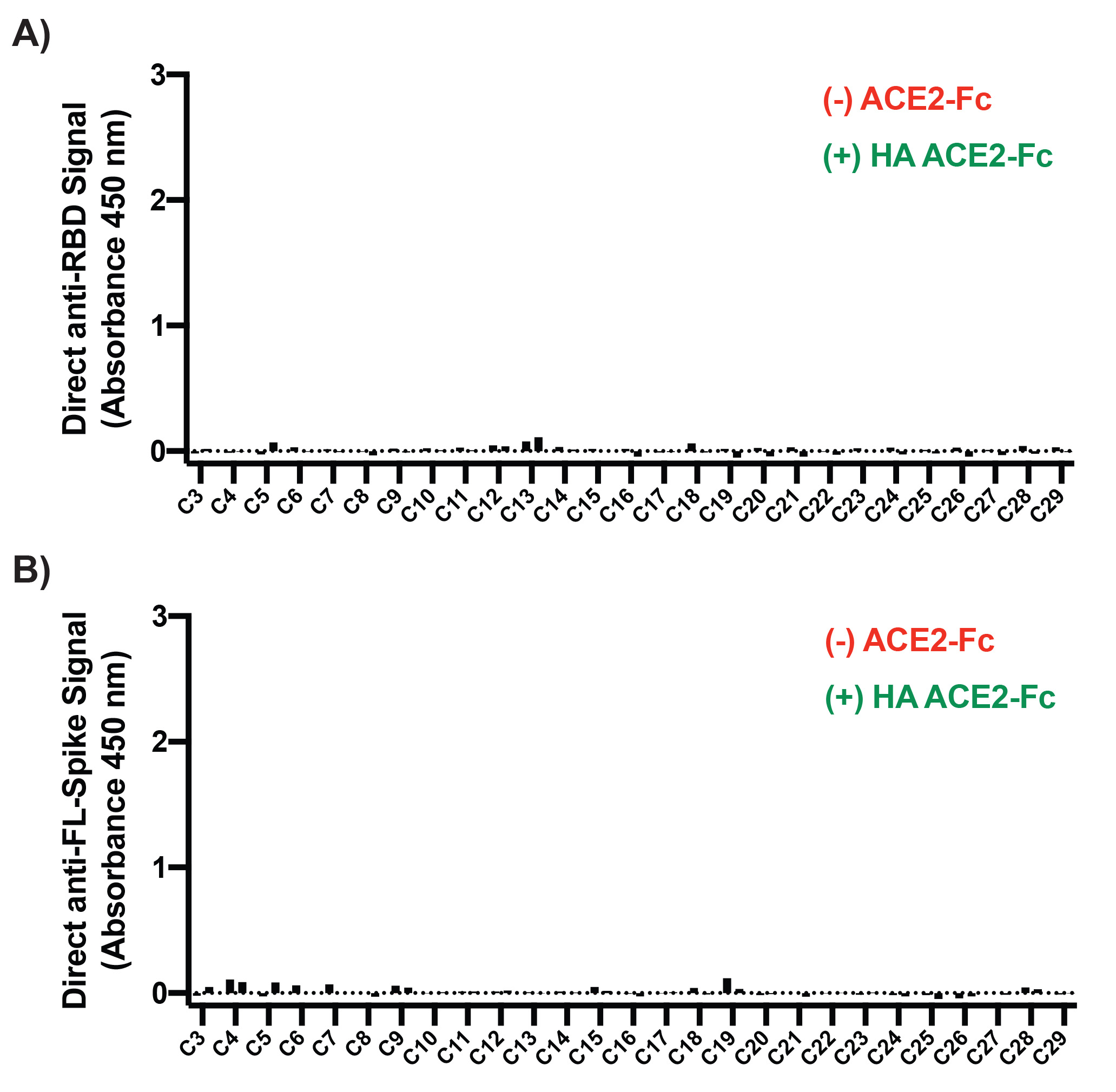

Supplement: FIG S9 [file mSphere.00802-20-sf009.jpg]

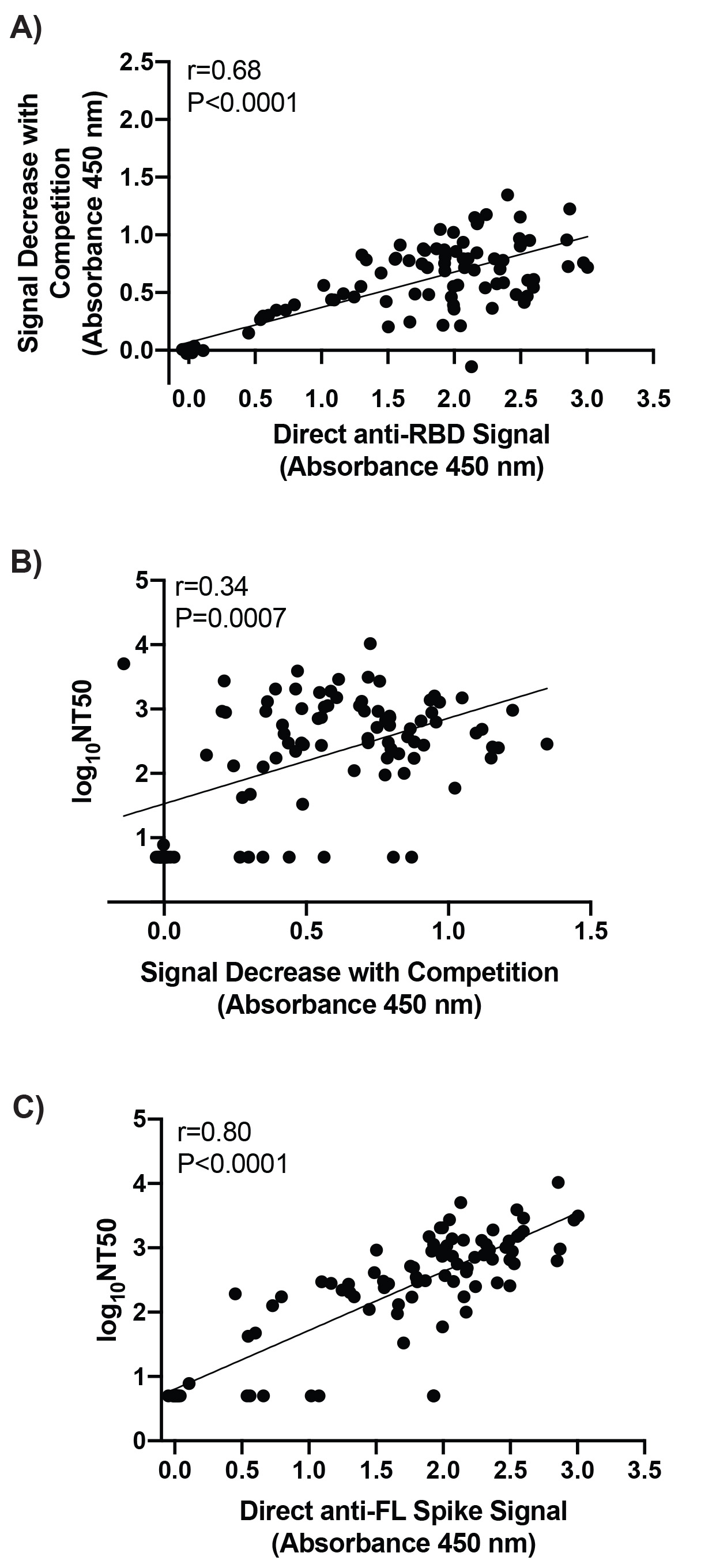

Supplement: FIG S10 [file mSphere.00802-20-sf010.jpg]
